# Emergent cholecystectomy in patients on antithrombotic therapy

**DOI:** 10.1038/s41598-020-67272-3

**Published:** 2020-06-22

**Authors:** Masashi Yoshimoto, Masayoshi Hioki, Hiroshi Sadamori, Kazuteru Monden, Satoshi Ohno, Norihisa Takakura

**Affiliations:** 1 Department of Gastroenterological Surgery, Fukuyama City Hospital, Fukuyama, Japan; 2 Department of Gastroenterological Surgery, Okayama University Hospital, Okayama, Japan

**Keywords:** Cholecystitis, Cholecystitis

## Abstract

The Tokyo Guidelines 2018 (TG18) recommend emergent cholecystectomy (EC) for acute cholecystitis. However, the number of patients on antithrombotic therapy (AT) has increased significantly, and no evidence has yet suggested that EC should be performed for acute cholecystitis in such patients. The aim of this study was to evaluate whether EC is as safe for patients on AT as for patients not on AT. We retrospectively analyzed patients who underwent EC from 2007 to 2018 at a single center. First, patients were divided into two groups according to the use of antithrombotic agents: AT; and no-AT. Second, the AT group was divided into three sub-groups according to the use of single antiplatelet therapy (SAPT), double antiplatelet therapy (DAPT), or anticoagulant with or without antiplatelet therapy (AC ± APT). We then evaluated outcomes of EC among all four groups. The primary outcome was 30- and 90- day mortality rate, and secondary outcomes were morbidity rate and surgical outcomes. A total of 478 patients were enrolled (AT, n = 123, no-AT, n = 355) patients. No differences in morbidity rate (6.5% vs. 3.7%, respectively; *P* = 0.203), 30-day mortality rate (1.6% vs. 1.4%, respectively; *P* = 1.0) or 90-day mortality rate (1.6% vs. 1.4%, respectively; *P* = 1.0) were evident between AT and no-AT groups. Between the no-AT and AC ± APT groups, a significant difference was seen in blood loss (10 mL vs. 114 mL, respectively; *P* = 0.017). Among the three AT sub-groups and the no-AT group, no differences were evident in morbidity rate (3.7% vs. 8.9% vs. 0% vs. 6.5%, respectively; *P* = 0.201) or 30-day mortality (1.4% vs. 0% vs. 0% vs. 4.3%, respectively; *P* = 0.351). No hemorrhagic or thrombotic morbidities were identified after EC in any group. In conclusion, EC for acute cholecystitis is as safe for patients on AT as for patients not on AT.

## Introduction

Acute cholecystitis is a common disease causing acute abdomen. The Tokyo Guidelines 2018 (TG18), as international practice guidelines for the management of patients with acute cholecystitis, provide diagnostic criteria, a severity grading scale, and a flowchart for the treatment of acute cholecystitis^1^. TG18 recommend early or emergent laparoscopic cholecystectomy (LC) as the first-choice treatment for Grade I (mild), Grade II (moderate) and Grade III (severe) acute cholecystitis under strict criteria^2^.

In recent years, the number of patients on antithrombotic therapy (AT) has increased significantly^3^. The beneficial effects of AT for patients with cardiovascular and cerebrovascular diseases have been established. Generally, in elective non-cardiac surgery, AT is interrupted or changed before surgery. Some reports have described the management of AT among patients undergoing invasive procedures^3^. Administration of reversal agents to patients taking anticoagulants has been recommended for emergent procedures. However, no evidence has yet been provided to suggest that emergent cholecystectomy (EC) should be performed for acute cholecystitis in patients on AT. Although early LC in patients with acute cholecystitis is well known to show good results^4,5^, clinicians often interrupt AT and do not perform EC until antithrombotic effects disappear. Delays in EC are associated with perioperative complications, longer hospital stay and higher hospital costs^6^. We have reported on the feasibility and safety of EC in patients on AT in a small number of cases^7^, but no evidence-based guidelines have yet been devised to define the optimal timing of EC in patients on AT for acute cholecystitis.

The aim of this study was to evaluate whether EC can be performed as safely in patients on AT as in patients not on AT.

## Methods

This was a retrospective study at a single center. Patients were diagnosed with acute cholecystitis in accordance with TG18 criteria^2^. All patients underwent computed tomography (CT) before emergent surgery. Magnetic resonance cholangiopancreatography (MRCP) was performed on patients who could keep sufficiently still (n = 438). In our institution, regardless of the severity of acute cholecystitis, when a patient can tolerate general anesthesia and agrees to emergent surgery, EC is performed as soon as possible. On the other hand, patients with severe respiratory failure, heart failure or valvular disease who cannot undergo general anesthesia instead receive gallbladder drainage.

We defined AT as the use of aspirin, warfarin, clopidogrel, cilostazol, ticlopidine, apixaban, dabigatran, edoxaban or rivaroxaban. If patients on warfarin therapy showed a prothrombin time-international normalized ratio (PT-INR) > 1.5, vitamin K or fresh frozen plasma (FFP) was injected before the surgery to reverse any anticoagulant effects. Other patients with acute cholecystitis underwent EC as soon as possible without discontinuation of AT.

Patients who had cholangitis or urinary tract infection were excluded from this investigation.

The following data were recorded: age, sex, presence of gallstones, severity of acute cholecystitis, time from onset to surgery, laboratory data (white blood cells (WBC); hemoglobin (Hb); platelets (Plt); PT-INR; C-reactive protein (CRP); serum total bilirubin (T-Bil); and creatinine (Cr)), American Society of Anesthesiologists (ASA) score, operation time, intraoperative blood loss, rate of intraoperative red blood cell (RBC) transfusion, rate of laparoscopic surgery, morbidity (Clavien-Dindo classification (CD) Grade≥ IIIa), length of hospital stay and 30- and 90-day mortality rate.

First, patients were divided into two groups: patients on AT; and patients not on AT. We compared the outcomes of EC between these two groups. Second, as antithrombotic effects may differ between agents, such as anticoagulant therapy (ACT) and antiplatelet therapy (APT), the AT group was divided into three subgroups: single APT (SAPT); double APT (DAPT); and anticoagulant with or without antiplatelet therapy (AC ± APT). We then compared outcomes of EC between the four groups (no-AT vs. SAPT vs. DAPT vs. AC ± APT). The primary outcomes were 30- and 90-day mortality rates, and secondary outcomes were morbidity rate and surgical outcomes.

All statistical analyses were performed using EZR version 1.33 (Saitama Medical Center, Jichi Medical University, Saitama, Japan), a graphical user interface for R (The R Foundation for Statistical Computing, Vienna, Austria). More precisely, EZR is a modified version of R Commander designed to add statistical functions frequently used in biostatistics^8^. Fisher’s exact test and Bonferroni correction were used for comparing categorical variables. Continuous variables were analyzed using the Mann-Whitney U test and multiple comparisons were performed using the Kruskal-Wallis and Steel-Dwass tests. Values of *P* < 0.05 were considered statistically significant.

All study protocols were approved by the ethics committee at Fukuyama City Hospital (permission number 268). The ethics committee determined this study was a retrospective observational study based on patient charts, and thus informed patient consent was therefore not required. All procedures in this study were performed in accordance with the relevant guidelines and regulations. Our study was carried out using the opt-out method of our institution website.

## Results

Between January 2007 and April 2018, a total of 478 patients (AT, n = 123; no-AT, n = 355) were enrolled. Table 1 shows the characteristics of overall cases. Significant differences in age (76 years vs. 69 years, respectively; *P* < 0.001), sex (74.0% vs. 62.2%, respectively; *P* = 0.021), presence of gallstones (76.4% vs. 86.4%, respectively; *P* = 0.015), severity grade I/III (28.5/35.8% vs. 47.6/13.0%, respectively; *P* < 0.001), ASA score ≥ 3 (58.5% vs. 34.1%, respectively; *P* < 0.001), Hb (12.8 mg/dl vs. 13.7 mg/dl, respectively; *P* < 0.001), Cr (0.9 mg/dl vs. 0.8 mg/dl, respectively; *P* < 0.001) and PT-INR (1.2 vs. 1.1, respectively; *P* < 0.001) were identified between the two groups.

**Table 1:** Overall patient characteristics.

Characteristics	Overall cases		
	AT (n = 123)	No-AT (n = 355)	*P*
Age, years∗	76 (47-94)	69 (18-95)	<0.001
Male sex (%)	91 (74.0)	221 (62.2)	0.021
Gallstone (%)	94 (76.4)	307 (86.4)	0.015
Onset-surgery (h)	48 (12-288)	48 (12-336)	0.842
Severity grade			
I (%)	35 (28.5)	169 (47.6)	<0.001
II (%)	44 (35.8)	140 (39.4)	0.519
III (%)	44 (35.8)	46 (13.0)	<0.001
ASA-PS ≥ 3 (%)	72 (58.5)	121 (34.1)	<0.001
Laboratory data			
WBC (×10^2^/µL)	127 (37-472)	114 (17-431)	0.021
Hb (mg/dL)	12.8 (8.3-16.8)	13.7 (6.6-17.6)	<0.001
Plt (×10^4^/µL)	18.5 (4.9-52.4)	20.3 (3.1-56.3)	0.066
T-Bil (mg/dL)	1.0 (0.2-14.6)	1.0 (0.2-8.5)	0.701
CRP (mg/dL)	12.1 (0.02-30.6)	6.9 (0.01-50.0)	0.001
Cr (mg/dL)	0.9 (0.30-9.05)	0.8 (0.4-10.1)	<0.001
PT-INR	1.2 (0.9-12.4)	1.1 (0.9-1.6)	<0.001

Data are presented as the percentage or median value of parameters [range].

Severity grade: According to the Tokyo Guidelines 2018 severity grading for acute cholecystitis.

ASA, American Society of Anesthesiologists; WBC, white blood cells; Hb, hemoglobin; Plt, platelets; T-Bil, total bilirubin; CRP, C-reactive protein; Cr, creatinine; PT-INR, prothrombin time-international normalized ratio.

Table 2 shows the details of the antithrombotic agents administered. Of the 123 patients in AT group, 41 had taken aspirin alone, 31 had taken warfarin alone and 17 patients had taken aspirin and clopidogrel. Many of the indications for AT were atrial fibrillation or ischemic heart disease (Table 3).

**Table 2:** Details of antithrombotic agents.

Agent	Patients (n = 123)
Aspirin	41
Warfarin	31
Aspirin + clopidogrel	17
Clopidogrel	8
Cilostazol	6
Apixaban	5
Aspirin + warfarin	4
Rivaroxaban	2
Edoxaban	1
Ticlopidine	1
Aspirin + apixaban	1
Aspirin + cilostazol	1
Aspirin + dabigatran	1
Aspirin + ticlopidine	1
Clopidogrel + cilostazol	1
Warfarin + cilostazol	1
Warfarin + clopidogrel	1

**Table 3:** Indications for antithrombotic therapy.

Comorbidity∗	Patients
AF	35
Ischemic heart disease	35
Ischemic stroke	35
CABG	7
Primary prevention of stroke	6
DVT	5
Mechanical valve replacement	3
PAD	3

∗Some patients showed multiple comorbidities.

AF, atrial fibrillation; CABG, coronary artery bypass grafting; DVT, deep vein thrombosis; PAD, peripheral arterial disease

Surgical outcomes for the AT and the no-AT groups are summarized in Table 4. Significant differences were seen in median blood loss (80 mL vs. 10 mL, respectively; *P* = 0.005), rate of RBC transfusion (8.1% vs. 2.5%, respectively; *P* = 0.013) and median hospital stay (7 days vs. 6 days, respectively; *P* < 0.001). No differences were evident in median operation time (110 min vs. 103 min, respectively; *P* = 0.212), rate of laparoscopic surgery (79.7% vs. 86.2%, respectively; *P* = 0.111), morbidity rate of CD Grade≥ IIIa (6.5% vs. 3.7%, respectively; *P* = 0.203), 30-day mortality rate (1.6% vs. 1.4%, respectively; *P* = 1.0) and 90-day mortality rate (1.6% vs. 1.4%, respectively; *P* = 1.0) between these two groups. AT was not a significant factor in 30-day mortality rate in Cox proportional hazards regression modeling (*P* = *0.838*).

**Table 4:** Perioperative outcomes for AT and no-AT groups.

	AT (n = 123)	No-AT (n = 355)	*P*
**Intraoperative**			
Operation time, min	110 (42-346)	103 (27-263)	0.212
Blood loss, mL	80 (0-2315)	10 (0-4880)	0.005
Laparoscopic surgery (%)	98 (79.7)	306 (86.2)	0.111
RBC transfusion (%)	10 (8.1)	9 (2.5)	0.013
**Postoperative**			
Morbidity (CD grade ≥IIIa) (%)	8 (6.5)	13 (3.7)	0.203
Length of stay, days	7 (2-55)	6 (1-82)	<0.001
30-day mortality (%)	2 (1.6)	5 (1.4)	1.0
90-day mortality (%)	2 (1.6)	5 (1.4)	1.0

Data are presented as the number of patients (%) or median [range].

CD, Clavien-Dindo; RBC, red blood cells.

**Figure 1:**
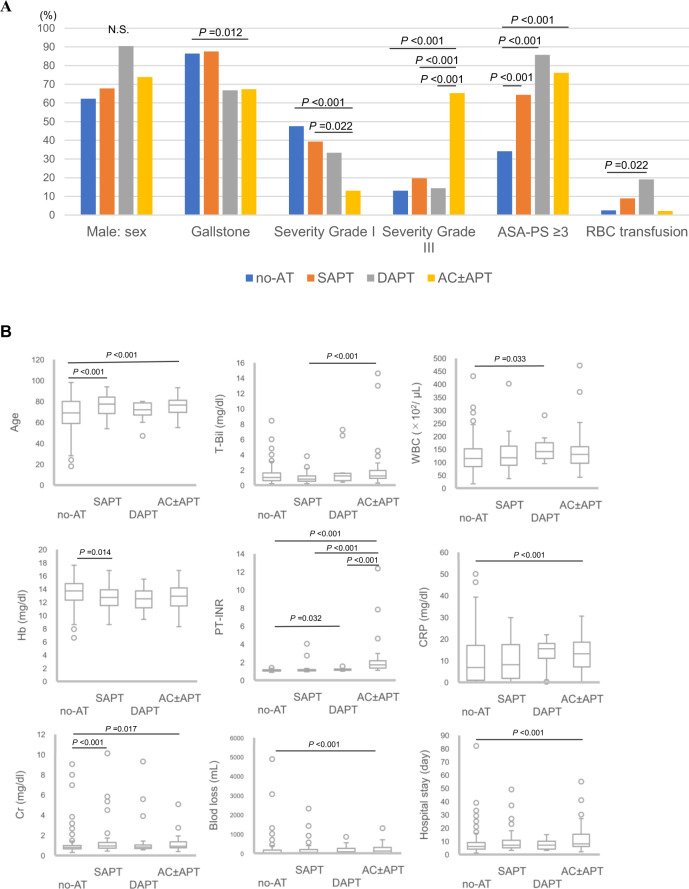
Multiple comparisons of characteristics and perioperative outcomes for no-AT, SAPT, DAPT, and AC±APT groups. (**A**) Fisher’s exact test and Bonferroni correction were used for comparing categorical variables. (**B**) Continuous variables were analyzed using the Mann-Whitney U test and multiple comparisons were performed using the Kruskal-Wallis and Steel-Dwass tests.

Table 5 shows the characteristics of the four groups (no-AT, SAPT, DAPT and AC ± APT). Surgical outcomes are summarized in Table 6. Figure 1 show multiple comparisons of characteristics and surgical outcomes. Patients in the AC ± ATP group included more patients with severity grade III (13% vs. 19.6% vs. 14.3% vs. 65.2%, respectively; *P* < 0.001) and higher PT-INR (1.1 vs. 1.2 vs. 1.2 vs. 1.7, respectively; *P* < 0.001) than patients in the other groups. Between the no-AT and AC ± APT groups, significant differences were seen in age (69 years vs. 79.5 years, respectively; *P* < 0.001), presence of gallstones (86.4% vs. 67.4%, respectively; *P* = 0.012), severity grade I (47.6% vs. 13.0%, respectively; *P* < 0.001), ASA-PS ≥ 3 (34.1% vs. 76.1%, respectively; *P* < 0.001), CRP (6.9 mg/dL vs. 13.1 mg/dL, respectively; *P* < 0.001), Cr (0.8 mg/dL vs. 0.9 mg/dL, respectively; *P* = 0.017), blood loss (10 mL vs. 114 mL, respectively; *P* = 0.017) and length of hospital stay (6 days vs. 8 days, respectively; *P* < 0.001). RBC transfusion rate was higher in the DAPT group than in the no-AT group (2.5% vs. 19%, respectively; *P* = 0.022). No differences were evident in the rate of morbidity and hospital mortality among the four groups.

**Table 5:** Patient characteristics of no-AT, SAPT, DAPT, and AC±APT groups.

Characteristics	No-AT (n = 355)	SAPT (n = 56)	DAPT (n = 21)	AC±APT (n = 46)	*P*
Age, years∗	69 (18-98)	77.5 (54-94)	72 (47-80)	76.5 (55-93)	<0.001
Male sex (%)	221 (62.2)	38 (67.8)	19 (90.4)	34 (73.9)	0.024
Gallstones (%)	307 (86.4)	49 (87.5)	14 (66.7)	31 (67.4)	0.002
Onset-surgery (h)	48 (12-288)	48 (12-336)	48 (12-144)	48 (12-72)	0.601
Severity grade (%)					
I (%)	169 (47.6)	22 (39.3)	7 (33.3)	6 (13.0)	<0.001
II (%)	140 (39.4)	23 (41.1)	11 (52.4)	10 (21.7)	0.052
III (%)	46 (13.0)	11 (19.6)	3 (14.3)	30 (65.2)	<0.001
ASA-PS ≥ 3 (%)	121 (34.1)	36 (64.3)	18 (85.7)	35 (76.1)	<0.001
Laboratory data					
WBC (×10^2^/ µL)	114 (17-431)	117 (37-402)	141 (95-281)	131 (42-472)	0.026
Hb (mg/dL)	13.7 (6,6-17.6)	12.7 (8.6-16.8)	12.5 (9.4-15.5)	12.9 (8.3-16.8)	<0.001
Plt (×10^4^/µL)	20.3 (3.1-56.3)	20.5 (8.1-52.4)	17.1 (9.7-35.2)	17.3 (4.9-37.9)	0.098
T-Bil (mg/dL)	1.0 (0.2-8.5)	0.8 (0.2-3.8)	1.2 (0.4-7.3)	1.2 (0.3-14.6)	0.015
CRP (mg/dL)	6.9 (0.01-50.0)	8.1 (0.02-29.8)	15.5 (0.23-17.9)	13.1 (0.3-30.6)	0.001
Cr (mg/dL)	0.8 (0.30-9.1)	0.9 (0.4-10.1)	0.8 (06-9.3)	0.9 (0.4-5.1)	<0.001
PT-INR	1.1 (0.9-1.8)	1.1 (0.9-4.1)	1.2 (1.0-1.6)	1.7 (1.1-2.1)	<0.001

Data are presented as the percentage or median value of parameters [range].

**Table 6:** Perioperative outcomes for no-AT, SAPT, DAPT, and AC±APT groups.

Characteristics	no-AT (n = 355)	SAPT (n = 56)	DAPT (n = 21)	AC ± APT (n = 46)	*P*
Intraoperative					
Operation time, min	103 (27-263)	109 (48-346)	114 (60-208)	115 (42-306)	0.477
Blood loss, mL	10 (0-4880)	50 (0-2315)	75 (0-850)	114 (0-1300)	0.019
Laparoscopic surgery (%)	306 (86.2)	48 (85.7)	17 (81.0)	33 (71.7)	0.086
RBC transfusion (%)	9 (2.5)	5 (8.9)	4 (19.0)	1 (2.2)	0.002
Postoperative					
Morbidity (CD grade ≥IIIa) (%)	13 (3.7)	5 (8.9)	0 (0)	3 (6.5)	0.201
MOF	4	0	0	0	
Respiratory failure	1	0	0	0	
Acute heart failure	0	0	0	1	
Bile leakage	3	2	0	1	
Intraabdominal abscess	3	1	0	0	
Cardiac arrythmia	1	1	0	0	
Pleural effusion	0	1	0	0	
NOMI	1	0	0	1	
Hemorrhagic complication	0	0	0	0	
Thrombotic complication	0	0	0	0	
Length of stay, days	6 (1-82)	7 (3-49)	7 (3-15)	8 (2-55)	0.002
30-day mortality (%)	5 (1.4)	0 (0)	0 (0)	2 (4.3)	0.351

Data are presented as the percentage or median value of parameters [range].

MOF, multiple organ failure; NOMI, non-occlusive mesenteric ischemia.

Multiple comparisons are shown in Fig. 1.

Several morbidities occurred in all groups (Table 6). Most morbidities, such as organ dysfunction or non-occlusive mesenteric ischemia (NOMI), were caused by sepsis. Three patients who experienced morbidity in the AC ± APT group underwent reversal of anticoagulants before EC. No hemorrhagic or thrombotic morbidities were identified after EC in any groups.

Seven deaths occurred among all patients: 2 in the AC ± APT group and 5 in the no-AT group. In the AC ± APT group, 1 patient died of NOMI caused by superior mesenteric artery stenosis due to arteriosclerosis on postoperative day (POD)19. The other died of acute exacerbation of chronic heart failure on POD7. In the no-AT group, 2 patients died because of multiple-organ failure (MOF) due to septic acute cholecystitis on POD1 and POD6. One patient with post-resuscitation encephalopathy who underwent EC, died because of aspiration pneumonia on POD6. Another patient experienced a severe asthma attack triggered by pneumonia and developed respiratory failure. He received steroid pulse therapy, but gastrointestinal perforation occurred and he died on POD25. The last patient died of Wegener’s granulomatosis presenting with severe acute respiratory failure on POD21.

## Discussion

TG18 provides diagnostic criteria, a severity grading scale and a flowchart for the management of acute cholecystitis^1,2^. They recommend early LC for Grade I, II and III acute cholecystitis under strict criteria. However optimal management for patients remains unclear.

Recommendations regarding the management of AT in patients undergoing invasive procedures have been reported. Those recommendations show the bleeding risk of procedures and the timing of cessation or resumption of AT during elective procedures^3^. When urgent or emergent procedures are required in patients taking warfarin, administration of reversal agents, such as vitamin K or FFP, is recommended. However, advice has been lacking regarding the management of antiplatelet therapy in patients undergoing emergent procedures.

For patients on AT, the most severe morbidity is perioperative hemorrhage and thrombosis. Patients on AT are clearly in a potentially hemorrhagic state. Our study showed significant differences in intraoperative blood loss and rate of RBC transfusion between the AT and no-AT groups. In an additional analysis, the AC ± APT group displayed a significantly higher PT-INR than other groups, and greater intraoperative blood loss than the no-AT group. In this study, all patients were checked for activated partial thromboplastin time and PT-INR before emergent surgery. The threshold at which the risk of bleeding increases is unclear, but the risk is assumed to remain unelevated when PT-INR is ≤1.5 and is assumed to be elevated for PT-INR > 2.0^9^. Thus, in patients on warfarin therapy with PT-INR > 1.5, we administered intravenous vitamin K or/and FFP for the reversal effects before surgery. All patients showing PT-INR > 1.5 were administered reversal agents before surgery. However, not all patients were checked to ensure PT-INR was <1.5 after administration of these reversal agents. Some patients might thus have displayed remnant anticoagulant effects during surgery. Significant differences were also seen in CRP concentrations. Risk of intraoperative bleeding depends on not only anticoagulant effects, but also the degree of gallbladder inflammation. Between the no-AT and AC ± APT groups, no significant differences were seen in rate of transfusion, morbidity or mortality. We thus considered the differences in blood loss as not representative of any problems in terms of postoperative course. The hemostatic ability of surgical devices has also improved over recent decades. Several reports have shown the useful of hemostatic ability of soft-coagulation system in hepatectomy or nephrectomy^10–12^. We often perform EC for acute cholecystitis using soft-coagulation systems. Intraoperative bleeding is also controllable using such surgical devices. We thus do not consider intraoperative bleeding as a reason to delay EC in patients on AT.

Re-initiation of AT is a major determinant of postoperative bleeding after emergency surgery. The duration of AT discontinuation should be as short as possible. We checked vital signs, laboratory parameters, abdominal ultrasonography and drainage fluid (if the patient had a drainage tube) after surgery for signs of postoperative bleeding. In addition, AT was resumed 24–48 h after surgery. Patients on warfarin therapy received bridging therapy with heparin. Using this method, we did not experience any instances of postoperative hemorrhage or thrombosis.

Several reports have described perioperative outcomes in patients with AT during elective surgery^13–17^. Some reports have described laparoscopic cholecystectomy in patients on antiplatelet therapy^18–21^. Randomized trials have also reported the effects of continued aspirin on various perioperative factors^22–24^. Devereaux et al. reported that perioperative use of aspirin increased major bleeding risk in patients undergoing noncardiac surgery (aspirin 4.6% vs. placebo 3.8%; *P* = 0.04). However, another report showed different results. This issue thus remains controversial.

AT can be classified into APT and ACT. APT is known as the most effective treatment for preventing cardiovascular and cerebrovascular events. Lifelong oral administration is thus recommended for patients who have experienced cardiovascular or cerebrovascular events^25–27^. Some reports have shown that discontinuation of APT among patients with coronary artery or cerebrovascular disease increases the risk of major adverse events^22^. In our study, many patients in the DAPT group underwent percutaneous coronary intervention (PCI) because of ischemic heart disease. Although no significant differences were seen in preoperative Hb or intraoperative blood loss between the no-AT and DAPT groups, the rate of transfusion was significantly higher in the DAPT group. Four patients who received intraoperative transfusion in the DAPT group had undergone open cholecystectomy because of severe inflammation around the gallbladder, and those patients suffered substantial blood loss (median, 433 mL). For DAPT cases in which open surgery is selected, more precise procedures and preparation of transfusion before surgery may be warranted. Importantly, our study showed no cardiovascular or cerebrovascular events after emergent surgery. This may be associated with no discontinuation of antiplatelet agents.

Anticoagulant therapy is used for patients with a mechanical heart valve, nonvalvular atrial fibrillation or venous thromboembolism. In elective procedures, bridging anticoagulation is often used for patients on warfarin therapy. The BRIDGE trial showed that forgoing bridging anticoagulation did not result in inferior results to perioperative bridging with low-molecular-weight heparin in terms of preventing arterial thromboembolism, but did decrease the risk of major bleeding^28^. In our study, warfarin therapy was restarted the day after surgery, and bridging anticoagulation was provided for 24–48 h after surgery. Bridging anticoagulation was continued until the INR reached the therapeutic range. Direct oral anticoagulant therapy was resumed 24–48 h after surgery without bridging anticoagulation. Whether bridging anticoagulation is beneficial thus remains uncertain. However, no hemorrhagic or thrombotic morbidity was encountered in our study. We thought that longer hospital stays in the AT ± APT group were due to management with anticoagulant drugs. According to TG18, Grade III cholecystitis is associated with hepatic dysfunction (PT-INR > 1.5). Patients displayed a high PT-INR because of warfarin therapy were classified as Grade III regardless of the degree of systemic inflammation. We considered this to be the reason why the AT group and AC ± APT group had more Grade III patients and higher PT-INR value than the other groups.

A few patients taking Direct Oral Anti Coagulants (DOACs) were included in this study. The half-lives of DOACs are shorter than that of warfarin^29^, and that of DOACs applied in this study ranged from 5 to 17 h^30^. In all cases, the interval time from last oral intake to emergent surgery was more than 12 h, so anticoagulant action was expected to decrease over time. In this study, since no parameters indicative of the anticoagulant effect of DOACs were examined, we cannot rule out the possibility that some anticoagulant effect of DOACs remained and affected outcomes for the AC ± APT group. We did not use new reversal agents, such as idarucizumab. The safety and utility of DOACs have been shown in comparison with warfarin, and increased use of DOAC is expected in the future^31^. And new reversal agents will play an increasingly role in emergent surgery.

In the AC ± APT group, 8 patients received combined APT and ACT. Several reports have evaluated bleeding risk in patients with combined APT and ACT^32–34^. However, in emergent surgery, the bleeding risk of combined therapy with reversed anticoagulant effect remains unclear. In our study, combined therapy did not increase intraoperative blood loss compared to anticoagulant monotherapy (100 mL vs. 130 mL, respectively; *P* = *0.699*), but the small sample size limits our ability to draw conclusions on how combined therapy affected outcomes for the AC ± APT group. Future work would ideally deal with these issues separately.

In our study, we had a case with significant blood loss. Patient with 4880 mL of the max blood loss in the no-AT group had Child-Pugh class C liver cirrhosis and had coagulopathy (PT-INR = 1.62) and thrombocytopenia (Plt = 7.7 × 10^4^/µL). Several reports showed safety and feasibility of cholecystectomy in patient with liver cirrhosis^35,36^. Median blood loss of 12 patients with liver cirrhosis in the no-AT group was 30 mL, and case with significant blood loss was unusual. But for patients with coagulopathy associated with liver cirrhosis, more precise indications, procedures and preparation of transfusion before surgery may be warranted as previous reports^35,36^. Even when the patient with this outlier were excluded, the analysis results were comparable.

Our study has certain limitations that need to be considered when interpreting the results. First, this study was a retrospective study design using a relatively small number of patients from a single center. This weakened validity of the statistical analysis and conclusions. However, given that few reports have considered EC on AT^18–21^, our findings appear valuable in that we evaluated more than 100 patients on multiple antithrombotic agents including antiplatelets and anticoagulants. A larger, well-designed, multicenter, randomized controlled trial is thus needed. Second, we treated various patients using antithrombotic agents as one group, further divided into four subgroups. However, antithrombotic effects differ between specific agents. Several agents were used in only a few cases, and further subgroup analysis is not feasible for this study. If more cases could be accumulated, we would be better able to clarify these situations. Third, although the criteria for EC indications or perioperative antithrombotic therapy managements were clear, no clear protocol was set for intraoperative transfusion or indications for conversion to open surgery. Patients on antithrombotic therapy display various comorbidities, and general condition during emergent surgery varies greatly from patient to patient. This makes it difficult to unify intraoperative management and we had to entrust intraoperative decisions to surgeons and anesthesiologists. Fourth, no cases in the present study had undergone gallbladder drainage, so we could not perform comparisons with elective cholecystectomy after gallbladder drainage.

In conclusion, emergent cholecystectomy appears safe to perform for acute cholecystitis in patients on AT thanks to the advances in surgical techniques and devices, and improvements to the perioperative management of AT.
